# Influence of energy supplementation on mitigating energy deficit and enhancing dairy performance in Holstein Friesian cows

**DOI:** 10.1007/s11250-025-04816-7

**Published:** 2025-12-24

**Authors:** Hany M. Gado, Dalia A. Ahmed, Mona M. M. Y. Elghandour, Pasquale De Palo, Abdelfattah Z. M. Salem

**Affiliations:** 1 Faculty of Agriculture, Ain Shams University, Hadayek Shoubra, P.O. Box 68, Cairo, 11241 Egypt; 2 Faculty of Agriculture, Cairo University, El-Gamaa Street/Orman, Giza, Cairo, 12613 Egypt; 3 Facultad de Medicina Veterinaria y Zootecnia, Universidad Autónoma del Estado México, Toluca, Estado de México México; 4 Dipartimento di Medicina Veterinaria, Università degli Studi di Bari, 70010 Valenzano, Bari, Italy; 5 Dipartimento di Scienze del Suolo, della Pianta e degli Alimenti (Di.S.S.P.A.), Università degli Studi di Bari, Via Giovanni Amendola, 165/a, 70126 Bari BA, Italy

**Keywords:** Dairy cattle, Energy supplementation, Milk production, Blood metabolites, Single-cell protein

## Abstract

This work sought to investigate the effects of different dosages of energy supplements (XE) on energy balance, dairy performance, and blood metabolites in Holstein Friesian cows. Ninety nursing Holstein Friesian cows were randomly distributed to three groups: Control (C), low dosage (0.1 kg/cow/day, XE1), and high dose (0.2 kg/cow/day, XE2). Over the course of one week, known as the acclimatization period, we gradually exposed the cows to the extra food. Using XE components glycerol and propylene glycol made via fermentation with single-cell protein, the treatment ran for eight weeks. Milk composition and its production, along with blood metabolites, were evaluate. The XE2 group significantly (*P* < 0.05) improved total digestible nutrients and net energy *versus * control group. Compared to other groups, cows in the XE2 group produced the most milk during the weeks; their relative increase from 29.5 L/day in week 1 to 31.5 L/day in week 8 is statistically significant (*P* < 0.05). Milk fat and protein notably (*P* < 0.05) increased in the high-dose group (XE2) by roughly 14% and 9%, respectively, *versus* control cows. Blood metabolites like beta-hydroxybutyrate, non-esterified fatty acids went down (*P* < 0.05), and urea nitrogen was nonsignificant, even though insulin and blood glucose levels went up, which means metabolic health and energy balance got better. Particularly in cows with outstanding production potential, the study found that introducing energy supplementation into dairy diets improved energy balance, milk yield, milk composition, and blood metabolites.

## Introduction

The success of lactation, along with metabolic health and overall productivity in dairy cows, is heavily influenced by energy balance (Martens 2023). Particularly in early lactation (Xu et al. 2020; Gross 2023), lactating cows have major metabolic demands arising from the energy required for milk production, which often causes negative energy balance. Under negative energy balance, cows use adipose tissue to meet their energy needs, which increases levels of non-esterified fatty acids and beta-hydroxybutyrate in the bloodstream, indicators linked to metabolic diseases such as ketosis (Yepes et al. 2019; Tessari et al. 2020). Improving both productivity and health depends on dairy cow energy intake being under control by dietary approaches (Martins et al. 2022). Dietary energy supplements are one possible fix for this issue. Adding dietary energy can help cows maintain their energy balance, reduce body fat mobilization, and enhance lactation performance (Chilliard 1992; Weber et al. 2013; Mekuriaw 2023). Following the Nutrient Requirements of Dairy Cattle guidelines, this supplementing approach emphasizes the need for exact energy formulation in maintaining cow health and optimizing milk output (NRC 2001). The protein level of their diet is favorably correlated with the production performance of dairy cows and dry matter intake (Law et al. 2009). High-protein diets are common in the dairy industry, used to improve milk output. However, when the diet’s protein level surpasses the need for milk production, it usually causes increased nitrogen excretion in urine and feces, therefore raising the risk of environmental pollution (Olmos Colmenero and Broderick 2006). Researchers are interested in alternative protein sources like insects, bacteria, and leftover plants because they can use renewable parts from agricultural waste without needing land (Spanoghe et al. 2021; Koukoumaki et al. 2024). Especially as single-cell protein, microorganisms provide a sustainable and environmentally friendly replacement for animal-derived proteins (Li et al. 2024). Derived from high-protein microbes, single-cell proteins can be used as animal feed with composition changed depending on the source (Ravindra 2000; Bratosin et al. 2021). Comprising up to 70% protein, they also include carbohydrates lipids, vitamins, and other minerals. The purpose of this study was to determine how low-dose and high-dose XE supplementation affected lactation performance, milk composition, and metabolic health in dairy cows under production. In particular, taking XE supplements changes the amount and make-up of milk over 8 weeks. It also changes blood metabolite profiles, which include glucose, non-esterified fatty acids, beta-hydroxybutyrate, and blood urea nitrogen. This study intends to offer important new perspectives on the possibilities of XE to improve the health and output of dairy cows and thereby balance energy consumption.

## Materials and methods

This work was approved by Ain Shams University’s Experimental Animal Care and Research Ethics Committee under the Agriculture Sector Committee with approval number 4-2024-10. Specifically, the Directions for Caring for Experimental Animals 2020, the use of animals in our research followed strictly the American Veterinary Medical Association (AVMA) recommendations for the Euthanasia of Animals 2020.

### XE product

The XE is a novel energy supplement meant to solve nursing dairy cow energy shortages. Bactizad Co. manufactures its XE product in Cairo, Egypt, using fermenting Saccharomyces cerevisiae and Bacillus sp. XE consists of 60% glycerol, 30% propylene glycol, and 10% single-cell protein (SCP). Under aerobic conditions, fermenting at 30 °C for 48 h used sugarcane molasses as the substrate. Centrifugation yielded SCP, lyophilized, then mixed with glycerol or propylene glycol. The energy content of the product is 16.5 MJ/kg. By increasing feed conversion efficiency through the fermentation in the XE formulation, animals can acquire more weight or generate more milk with less feed consumption. Single-cell microorganisms may be able to improve the total energy content of XE through fermentation. A company called Bactizad Co. confirms XE’s formula by using Kjeldahl analysis (65% of the SCP fraction) and HPLC (glycerol/propyleneglycol).

### Experimental groups, feedings and treatments

A total of 90 lactating Holstein Friesian cows in their second lactation season from a private farm in Noubaria, Egypt, were randomly distributed into three experimental groups of thirty cows each: Control, low dose XE (XE1), and high dose XE (XE2). To reduce uncertainty, cows were paired according to age, parity, and days in milk. Cows were block‑randomized by parity (second lactation) and days in milk ( 45 ± 7 at enrollment) with a mean milk yield of 28 ± 0.75 L/cow/day using a computer‑generated sequence with 1:1:1 allocation to Control, XE1, and XE2. Pairing during enrollment was used only to define blocks; analysis treats cow as the experimental unit. Mean temperature humidity index (THI) during the trial was 67 (range 64–70), thermoneutral for cows. No differences among groups, as all cows were in the same controlled environment. Serving as experimental units for eight weeks, all cows were maintained separately in a contemporary dairy facility furnished with rubber-matted flooring, automatic ventilation, and unlimited access to water and feed. Individual stalls of 4 m by 2 m housed cows with sand bedding. They had access to the milking parlor twice daily, where they maintained a temperature between 18 and 22 °C. Standard management guidelines were followed, including regular health checks and twice-daily milking. The control group of dairy cows received a normal NRC-compliant diet, tailored to their dietary needs. To attain homogeneity, feed components were mixed in a feed mixer and evaluated in compliance with the American Association of Cereal Chemists’ (AACC 2023) criteria. The control diet enriched with XE at rates of 20% (0.1 kg/cow/day) and 40% (0.2 kg/cow/day), respectively, was given to cows in the XE1 and XE2 groups. The energy deficit was calculated as follows: NRC (2001) computations indicate that energy intake (MJ/day) - energy required for maintenance and lactation (MJ/day) is every cow’s daily diet was tailored to fit the particular treatments described in Table 1. Every cow’s total mixed ration (TMR) was weighed daily to determine dry matter intake (DMI), and then the orts (uneaten feed) gathered the following morning were deducted. To determine the DM content, feed samples were dried at 60 °C for 48 h; dry matter intake (*i.e.*, DMI) was then calculated as the difference between the given feed’s dry weight and orts.

**Table 1:** Feed composition and feed analysis for the experimental treatments

Ingredient (kg/cow/day)	C	XE1	XE2
Corn silage	12	12	12
Alfalfa hay	3	3	3
Soybean meal	2	2	2
Corn grain	6.	5.5	5
XE supplement	0	0.1	0.2
Mineral premix	0.5	0.5	0.5
Total	23.5	23.1	22.7
Nutrient (g/kg)			
Dry matter	850	850	850
Crude protein	160	160	160
Neutral detergent fiber	350	350	350
Acid detergent fiber	250	250	250
Hemicellulose	100	100	100
Crude fat	30	30	30
Ash	80	80	80
Calcium	12	12	12
Phosphorus	8	8	8
Potassium	20	20	20
Sodium	5	5	5
Magnesium	3	3	3
Sulfur	1.5	1.5	1.5
Zinc	25	25	25
Copper	10	10	10
Manganese	40	40	40
Selenium	0.3	0.3	0.3
Iodine	0.2	0.2	0.2
Vitamin A (IU/kg)	20,000	20,000	20,000
Vitamin D (IU/kg)	2,000	2,000	2,000
Vitamin E (IU/kg)	100	100	100

All diets were analyzed for nutrient composition, ensuring consistency across the three treatment groups

C: control group, XE1: low dose of XE, XE2: high dose of XE

All energy values of feeds are explicitly on a dry‑matter basis and calculated using the following equations:

- TDN diet = Σ(wj×TDNj).
- ME diet = Σ(wj×MEj).
- NE diet = Σ(wj×NEj).

### Milk production and composition

Matching cows for age (3.2 ± 0.5 years), parity (second lactation), and days in milk ( 45 ± 7 days at the start of the trial). The cow’s daily milk output was recorded on a computerized milking system. Using a milk analyzer, milk samples were gathered biweekly for analysis of fat, protein, and lactose percentages. The samples were investigated using AOAC (2023). As such, every cow’s milk was taken at regular two-week intervals during the study. Four milk samples from each cow throughout the trial were collected, taking into account the eight-week data range. Since cows are milked bi-daily, milk samples were gathered in the morning and afternoon during regular milking operations and mixed to reflect a daily sample. This method ensures exact milk composition during the experiment. The sample size per group at each week was 30, without missing any samples.

### Blood metabolites

Blood samples were drawn weekly from the jugular vein of each cow. Samples of blood were centrifuged to extract the serum, which was then stored at -20 °C until analysis. Glucose, non-esterified fatty acids and beta-hydroxybutyrate, and blood urea nitrogen were determined using a colorimetric enzymatic assay (Bartlett and Gilbert 2014).

### Body condition scoring

Monthly evaluations of body condition scores using a standardized scoring method helped to ascertain the cows’ energy level in line with the USDA Body Condition Scoring method (2001). The Body Condition Score ran from 1 to 5:

One = emaciated, two = thin, three = moderate, four = good, and five = obese were the assigned category values.

### Statistical analysis

SPSS version 2023 was used for statistical analysis of the data. We used ANOVA to examine the means of milk yield, milk composition, and blood metabolites among the three groups—Control, XE1, and XE2. A Linear Mixed-Effects Model was used to look at longitudinal data (milk and blood metabolites) to see how things changed within the cows over time and how they differed between groups. The individual cow within each treatment was used as an experimental unit, and the differences among treatment groups were estimated using the following model:

$$\mathrm{Yijt}=\mu+\mathrm{G}_\mathrm{i}+\mathrm{C}_\mathrm{j}+\mathrm{T}_\mathrm{t}+\mathrm{(G}_\mathrm{i}\times\mathrm{T}_\mathrm{t}\mathrm{)}+\mathrm{e}_\mathrm{ijt}$$

whereas G is the fixed effect of the group, C: Random effect of Cow, T: Fixed effect of Time, G×T: Interaction effect between Group and Time.

We used analysis of variance and Tukey’s test to find differences between the treatments that were statistically significant (*P* < 0.05).

## Results

### Energy utilization

Whereas the cows in the XE1 and XE2 groups consumed an additional 1.65 and 3.30 MJ/day, respectively, depending on XE, the cows in the control group consumed a total of 160 MJ/day, following a normal NRC diet. Of all the groups, the XE2 group had the highest energy set aside for productive activities, that is, milk output. While both metabolizable energy (ME) and net energy (NE) rose by 0.6 MJ/kg in the same comparison, total digestible nutrients (TDN) surged by almost 40 g/kg from the control group to the high-dose XE group (Table 2). The control group ate 23.5 kg/day, while the XE1 and XE2 groups ate 23.1 kg/day and 22.7 kg/day, respectively. There was a lot of difference (*P* < 0.05) in the amount of dry matter (DMI) that each treatment group ate. If the DMI went down in the groups that were given XE, it could mean that the higher energy density of the added foods made the feed more efficient. Body condition scores (BCS) went up significantly (*P* < 0.05) with XE supplementation, going from 2.5 in the control group to 2.8 and 3.0 in XE1 and XE2, respectively. This increase in BCS suggests reduced fat mobilization in supplemented cows and a better energy economy.

**Table 2:** Effect of energy supplementation on daily energy parameters of lactating dairy cows

	C	XE1	XE2	SEM	*P*- value
DMI (kg/day)	23.5^ᶜ^	23.1^ᵇ^	22.7^ᵃ^	0.21	0.048
BCS (1–5 scale)	2.5^ᶜ^	2.8^ᵇ^	3.0^ᵃ^	0.03	0.045
Energy deficit (MJ/day)	20^a^	18.35^b^	16.7^c^	0.77	0.048
Supplemented energy (MJ/day)	0^c^	1.65^b^	3.3^a^	0.41	0.047
Energy available (MJ/day)	140^c^	143.3^b^	146.6^a^	0.64	0.046
TDN (g/kg)	700^c^	720^b^	740^a^	2.11	0.045
ME (MJ/kg)	11.5^b^	11.8^b^	12.1^a^	0.032	0.049
NE (MJ/kg)	9^c^	9.3^b^	9.6^a^	0.023	0.048

Numbers with different letters (a, b,c) in each row differ significantly at P < (0.05)

C: control group, XE1: low dose of XE, XE2: high dose of XE, TDN: Total digestible nutrients, ME: Metabolizable energy, NE: Net energy, DMI: Dry mater intake, BCS: Body condition score

### Milk production and composition

**Fig. 1:**
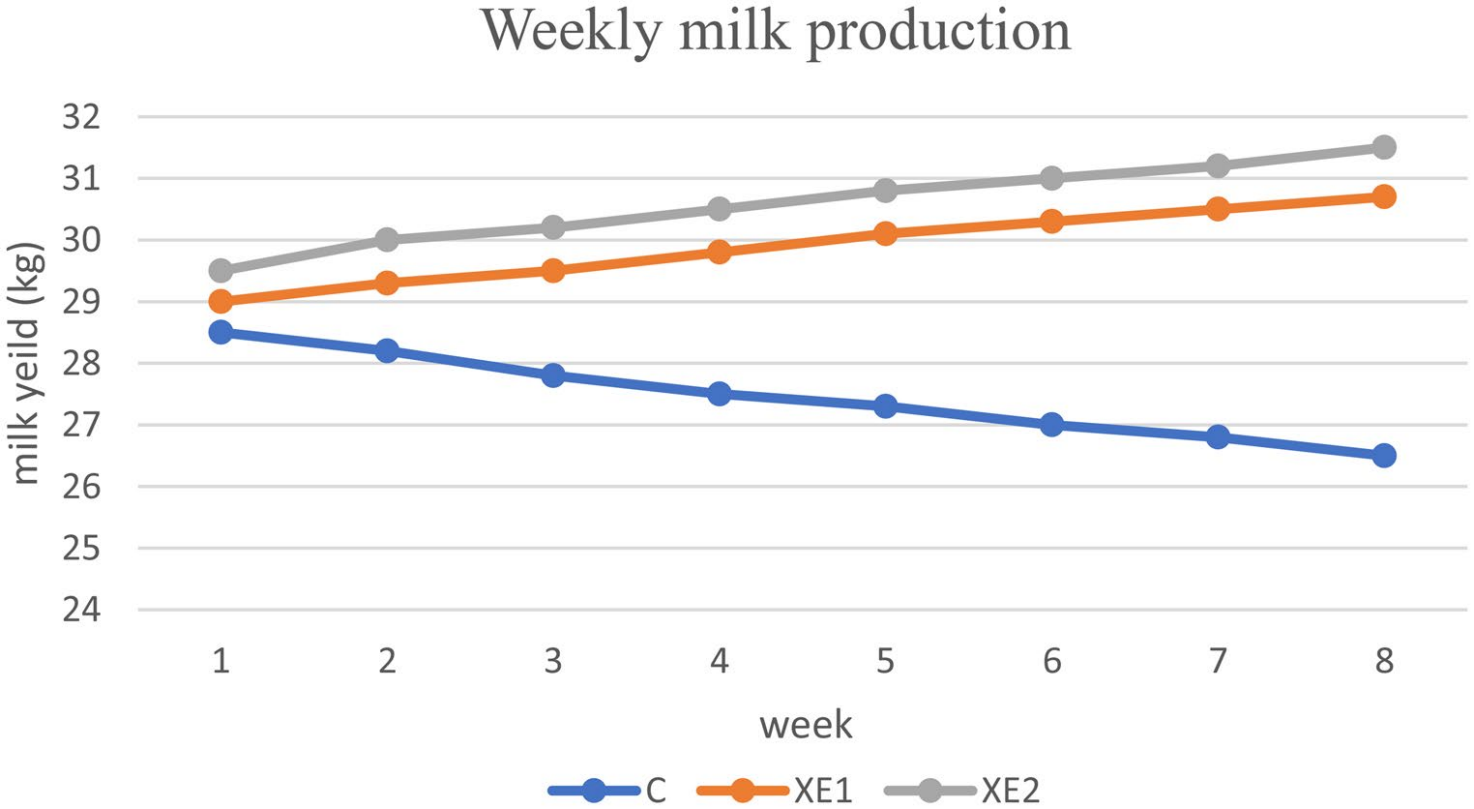
Effect of energy supplementation on weekly milk production, daily milk production (L/cow/day) during the experiment

Over the eight weeks, the milk output of cows in the control group steadily dropped by around 2 L per cow. As shown in Table 3; Fig. 1, milk production went up significantly (*P* < 0.05) in both groups. It went up by over 6.8%, or nearly 1.7 L/cow, in the XE1 group and almost 2 L/cow in the XE2 group. By 0.5%, the XE2 group’s lipid level was notably raised (*P* < 0.05) from the control group. Protein concentration thus increases from 3.3% in the control group to 3.6% in the high-dose group (Table 4). However, Table (5) shows exact P-values for primary outcomes (milk yield, non-esterified fatty acids and beta-hydroxybutyrate) mean differences with 95% CIs.

**Table 3:** Effect of energy supplementation and weekly production on milk production (L/cow/day) during the experiment

Week	C	XE1	XE2	Mean
1	28.5^l^	29k	29.5ij	29
2	28.2^lm^	29.3^jk^	30^h^	29.2
3	27.8^mn^	29.5^ij^	30.2^fgh^	29.2
4	27.5^no^	29.8^hi^	30.5^def^	29.3
5	27.3^o^	30.1^fgh^	30.8^bcd^	29.4
6	27.0^op^	30.3^efg^	31^bc^	29.4
7	26.8^pq^	30.5^def^	31.2^ab^	29.5
8	26.5^q^	30.7^cde^	31.5^a^	29.6
Mean	47.5	29.9	30.6	
SEM	0.19	0.19	0.19	

Numbers with different letters (a, b,c) in each row differ significantly at P < (0.05)

C: control group, XE1: low dose of XE, XE2: high dose of XE

**Table 4:** Effect of energy supplementation on milk parameters (%) in lactating dairy cows

	C	XE1	XE2	SEM	*P*- value
Milk fat	3.5^c^	3.8^b^	4^a^	0.026	0.049
Milk protein	3.3^c^	3.5^b^	3.6^a^	0.016	0.048
Milk lactose	4.9	5	5	0.006	N.S.

Numbers with different letters (a, b,c) in each row differ significantly at P < (0.05)

C: control group, XE1: low dose of XE, XE2: high dose of XE

**Table 5:** Exact P-values for primary outcomes (milk yield, non-esterified fatty acids (NEFA) and beta-hydroxybutyrate (BHBA)) mean differences with 95% CIs

Contrast	Estimate (Δ)	95% CI	Adjusted *P*
milk_yield_L_/_d			
XE1 − Control	2.166	[0.429, 3.903]	0.029
XE2 − Control	4.073	[2.508, 5.638]	< 0.001
XE2 − XE1	1.907	[0.170, 3.644]	0.031
NEFA_mmol_L			
XE1 − Control	-0.061	[-0.114, -0.007]	0.055
XE2 − Control	-0.111	[-0.173, -0.049]	0.001
XE2 − XE1	-0.051	[-0.112, 0.011]	0.109
BHBA_mmol_L			
XE1 − Control	-0.111	[-0.176, -0.046]	0.002
XE2 − Control	-0.181	[-0.245, -0.117]	< 0.001
XE2 − XE1	-0.070	[-0.148, 0.009]	0.081

C: control group, XE1: low dose of XE, XE2: high dose of XE

### Blood metabolites

**Fig. 2:**
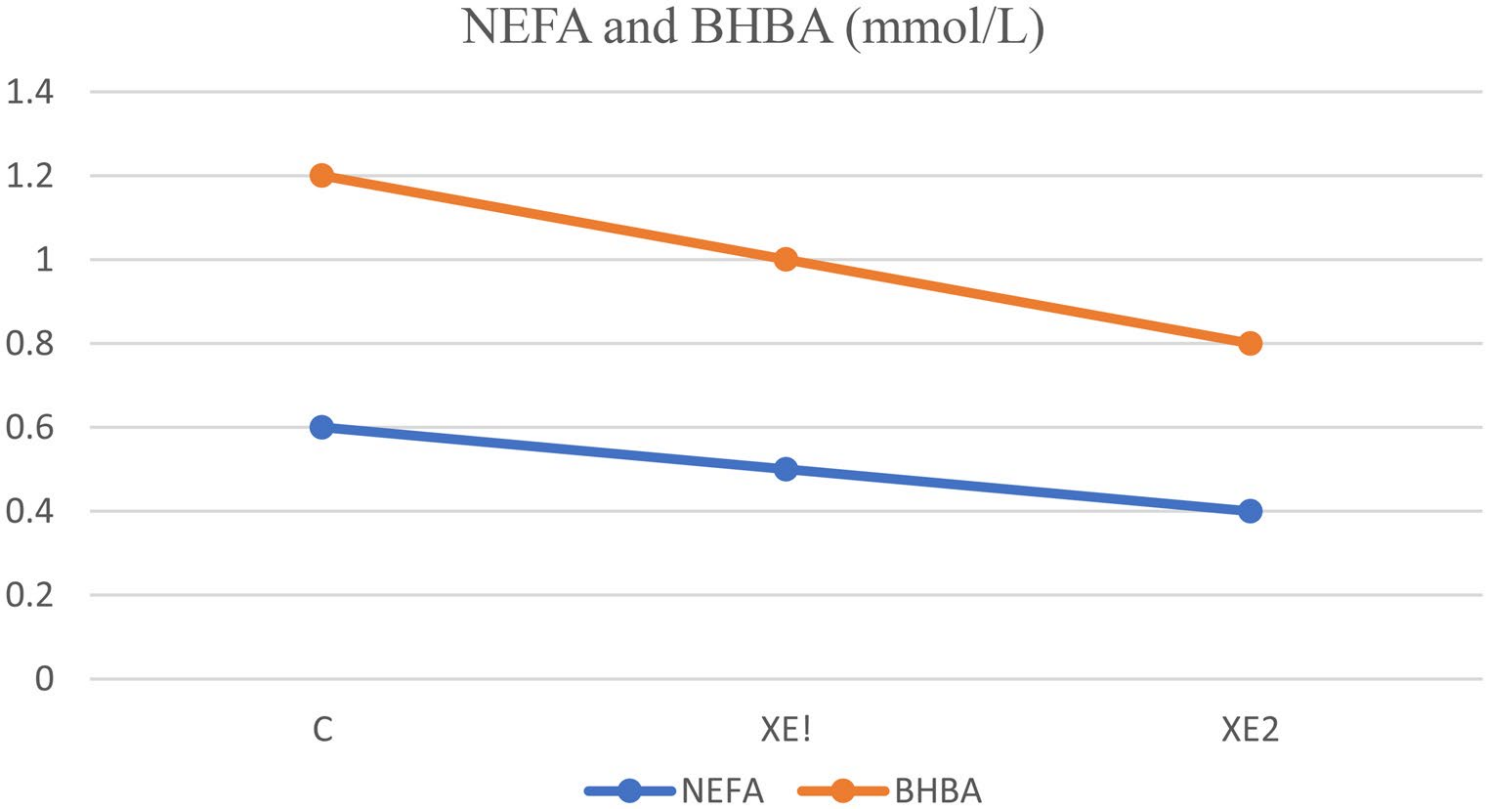
Effect of energy supplementation on non-esterified fatty acids (NEFA) and beta-hydroxybutyrate (BHBA) by group (mean ± SEM)

Table 6 shows how XE supplementation affects blood metabolites. By 10 mg/dL and 1.5 µIU/mL, respectively, glucose and insulin concentrations rose noticeably in the XE2 group from the control group. However, non-esterified fatty acids and beta-hydroxybutyrate levels dropped significantly (*P* < 0.05) in the XE2 group compared to the control group, by 0.2 and 0.4 mmol/L, respectively. This showed better metabolic health and energy balance. In the XE2 group, cholesterol and triglyceride levels were considerably reduced (*P* < 0.05) by about 15 and 10 mg/dL, respectively, compared to the control group. Reduced from 7.8 µg/dL in the control group to 6.3 µg/dL in the XE2 group, cortisol levels, a stress hormone, dropped (Table 6; Fig. 2).

**Table 6:** Impact of energy supplementation on blood metabolites, enzyme activities, and hormonal profiles in lactating dairy cows

Unit	C	XE1	XE2	SEM	*P*-value
Glucose (mg/dL)	55^c^	60^b^	65^a^	0.526	0.049
NEFA (mmol/L)	0.6^a^	0.5^b^	0.4^c^	0.011	0.048
BHBA (mmol/L)	1.2^a^	1^b^	0.8^c^	0.021	0.047
Blood urea nitrogen (mg/dL)	15	14	13	0.105	N.S.
Total cholesterol (mg/dL)	150^a^	140^b^	135^c^	2.145	0.046
Triglycerides (mg/dL)	70^a^	65^b^	60^c^	1.526	0.045
Insulin (µIU/mL)	15.5^c^	16.2^b^	17^a^	0.322	0.049
Cortisol (µg/dL)	7.8^a^	6.9^b^	6.3^c^	0.164	0.048
AST (U/L)	35^a^	32^b^	30^c^	0.823	0.047
ALT (U/L)	45^a^	40^b^	38^b^	1.145	0.046
Creatinine (mg/dL)	1.2	1.1	1	0.024	N.S.

Numbers with different letters (a, b,c) in each column differ significantly at P < (0.05)

C: control group, XE1: low dose of XE, XE2: high dose of XE, NEFA: Non-esterified fatty acids, BHBA: Beta-hydroxybutyrate, AST: Aspartate transaminase

ALT: Alanine aminotransferase

## Discussion

A negative energy balance in dairy cows increases the likelihood of ketosis development. In dairy cows, ketosis compromises milk production, metabolic health, and reproductive ability (Zhang et al. 2020; Martens 2023).

### Energy values

Following a conventional NRC diet, cows in the control group ate 160 MJ/day; those in the XE1 and XE2 groups ate an extra 1.65 and 3.30 MJ/day, respectively, since XE was included in their diets. Through components like glycerol and propylene glycol, which are quickly metabolized and essentially meet the cows’ energy needs during early lactation, coinciding with peak milk production and addressing their immediate energy needs during energy-deficient conditions, this inclusion offers a concentrated energy source (Van Knegsel et al. 2007). The fact that the dry matter intake (DMI) dropped more in the XE-supplemented groups (23.1 kg/day for XE1 and 22.7 kg/day for XE2) than in the control group (23.5 kg/day) suggests that the feed was more efficient, which is probably because the supplemented diets had more energy. This is in line with the results of DeFrain et al. (2004), which showed that while retaining or improving energy balance and milk supply, energy-dense supplements may lower dry matter intake. This extra energy demand would mean early lactation cows often find a negative energy balance (NEB), which is typified by insufficient energy intake that causes physiological reserves to mobilize. Long-term negative energy balance could hurt milk production, reproductive health, and the chance of metabolic disorders like ketosis (Grummer 2008; Yepes et al. 2019; Tessari et al. 2020).

The decrease in deficit in the XE2 group indicates the effect of XE on overall energy intake and implies that supplemented energy is helping the cows to more efficiently meet their metabolic energy needs. Particularly in early lactation, when the energy needs for milk production exceed their intake capacity, lactating cows can find an energy shortfall (Drackley et al. 2001). Given their use of fat and muscle for compensation, the limited energy supply implies that the cows would suffer from lower milk output and maybe worsen in body condition (Roche et al. 2018). The fact that body condition scores (BCS) increased from 2.5 (control) to 3.0 (XE2) shows that XE supplementation improved energy partitioning, which meant that less fat had to be mobilized. This is consistent with Roche et al. (2009), who found that energy supplements improve body reserves, which are vital for general health and reproductive function in nursing cows. The XE groups may not develop major metabolic problems because they have a smaller energy deficit. This means that they don’t need to mobilize as much body fat, which lowers the risk of ketosis. These results are similar to those found by Oetzel (2004), who found that adding propylene glycol can boost energy levels and lower the risk of ketosis during early lactation, which leads to more milk production.

The XE2 group had higher amounts of total digestible nutrients than the control. This was because the highly digestible energy sources in XE made the feed easier to digest and full of energy. Quickly absorbed and converted into glucose by gluconeogenesis, glycerol can significantly raise the energy availability for milk production (DeFrain et al. 2004). As a gluconeogenic precursor, propylene glycol increases energy availability and helps to offset the energy deficit during lactation (Oetzel 2004). The fact that XE-supplemented groups had lower dry matter intake and higher body condition scores shows that adding energy can help with two things: improving energy balance and making feed more efficient. Particularly in high-yielding dairy cows, these results show that XE not only increases milk production but also reduces the metabolic stress connected with negative energy balance.

### Milk production and composition

Particularly in early lactation, when dairy cows suffer an energy deficit due to the significant energy needs for milk synthesis, the energy composition of the diet strongly influences milk yield in these animals. Over the eight weeks, the milk output of the control cows routinely drops by about two liters per cow. This declining tendency could be explained by the lack of additional energy, which would have limited the cows’ ability to sustain high milk output levels. Maintaining milk yield depends on energy balance; in the absence of additional energy to offset the shortfall, cows often consume body reserves, therefore lowering production (Marshall et al. 2022). Over eight weeks, cows given XE showed a progressive rise in milk yield of roughly 1.7 and 2 L/cow (XE1 and XE2, respectively). Added the XE to the meals provided an extra 1.65 MJ/day of energy, which seemed to improve milk output *versus*  control group. The interaction of weeks and treatments emphasizes how cumulative the influence of energy supplements is over time. This emphasizes the need to control energy balance, especially in early lactation when cows are more prone to energy shortages. These results line up with studies showing that improving the energy balance of nursing cows, especially high-yielding animals, maximizes milk production (NRC 2001). The 6.8% increase in milk output correlates with Oetzel’s (2004) finding that propylene glycol addition improved glucose availability, reducing non-esterified fatty acids (NEFA) mobilization.

In the control group, milk’s fat level was 3.5%; in the high-dose group, it increased to 4%. Including XE into the diet raised the milk’s protein amount from 3.3% to 3.6%. The fast gluconeogenesis of glycerol (DeFrain et al. 2004) and the amino acid content of single-cell proteins almost certainly helped make more milk protein. A better energy balance is responsible for the increase in milk fat and protein since cows with enough energy reserves distribute more nutrients toward milk fat synthesis and production. Furthermore, energy supplements provide the required minerals, thereby improving the milk quality (Tessari et al. 2020; Wu and Satter 2000).

The higher BCS of cows in the high-dose XE group indicates better energy level and body condition. With a *P* < 0.05, XE2 (3.0) relative to the control (2.5), BCS showed a significant increase in energy state. From 2.5 in the control group to 3 in the high-dose cows, the improvement in BCS indicates an improved energy status, physical condition, and a lowered risk of metabolic disorders. These findings complement those of Roche et al. (2009), who found that cows fed extra calories show improved body reserves, therefore affecting general health and reproductive function.

### Blood metabolites

The higher glucose and insulin levels in the XE-supplemented groups indicate better energy equilibrium, which helps to increase metabolic activity and milk supply. Higher insulin levels mean that the XE supplement improves glucose absorption by cells, therefore improving energy availability and reducing the need for fat mobilization. This line of agreement with the glucose data suggests higher levels resulting from increased insulin activity. Comparable findings showing that dietary energy supplements improved glucose use and insulin sensitivity in stressed mice were reported by Zhang et al. (2020).

The drop in NEFA levels in the XE1 and XE2 groups emphasizes how well energy supplements help to reduce body fat mobilization. This is a vital sign of improved energy balance and metabolic health; Grummer (1993) proposed that higher NEFA levels often indicate cows are mobilizing adipose tissue to meet energy needs. Crucially, a sign of ketosis, beta-hydroxybutyrate (BHBA) levels dropped from 1.2 mmol/L in the control group to 0.8 mmol/L in the high-dose XE group. According to Oetzel (2004), this drop indicates a lower likelihood of subclinical ketosis in cows given XE since energy supplementation helps to prevent too high fat mobilization and ketone body generation, which are necessary to maintain metabolic health during early lactation. These trends line up with what Conte et al. (2018) found: by improving general energy balance, energy supplements reduce lipolysis and ketogenesis in cattle. Reiterating the idea that XE supplementation helps to preserve energy balance, Pascottini et al. (2020) found that lower concentrations of NEFA and BHBA correspond with lower metabolic stress.

The XE2 group, lower cholesterol levels suggested better lipid metabolism and maybe more hepatic activity. The drop in levels fits with the idea that XE supplements improved liver function by lowering oxidative stress. This is because oxidative damage can mess up the liver’s lipid metabolism. Triglycerides that were lower in the XE2 group showed that they were eating more fat, which makes mitochondria work better and burn more fat.

A stress hormone, cortisol, dropped in the high-dose group (approximately 1.5 µg/dL) relative to the control group. For stressed animals, especially, reducing cortisol levels is quite beneficial since continuous high levels could weaken immune capacity and general health. The lower cortisol levels suggested that the XE supplement helps the animals to reduce the effects of stress. The lower cortisol level could show that the supplement can help reduce the physiological stress reaction, which helps to reduce oxidative stress and inflammation (Xiao et al. 2021).

When XE was added to the diet, levels of creatinine, blood urea nitrogen, and liver enzymes (aspartate transaminase and slanine aminotransferase) went down. This suggests that XE had a protective effect on the kidneys and liver. As stated by Akosman et al. (2018), the XE supplement’s properties may protect renal cells from oxidative damage, hence improving kidney function. Energy-enhancing supplements, according to Kadzere et al. (2013), can reduce oxidative damage to the kidneys and liver, therefore improving organ performance in stressed mice.

The results show that XE supplements improved cholesterol and glucose metabolism, reduced stress, and protected kidney and liver functions. While increased insulin levels and lower cortisol indicate more energy intake and a reduced stress response, the declines in cholesterol, triglycerides, and liver enzymes indicate greater metabolic efficiency and reduced oxidative stress. The aforementioned advantages match the results of Akosman et al. (2018), Zhang et al. (2020), and Pinheiro et al. (2023), all of which show how well energy-dense pills improve animal health and metabolic performance.

## Conclusions

The results of the study showed that adding a high dosage of energy used (a commercial product including glycerol, propylene glycol, and a single-cell protein formula) to animal diets raised milk production by about 6.8%. It also changed milk composition; milk fat increased by roughly 14% while protein increased by 9% from the control group. It also lowered non-esterified fatty acids and beta-hydroxybutyrate levels, which stopped fat from being mobilized and prevented ketosis. This shows that energy supplements are good for milk protein and fat, especially in cows that produce a lot of milk. It also improved general animal health.

## Data Availability

Not applicable.
